# Effective modulation of CD4^+^CD25^+high^ regulatory T and NK cells in malignant patients by combination of interferon-α and interleukin-2

**DOI:** 10.1007/s00262-012-1297-2

**Published:** 2012-06-22

**Authors:** Guangxian Liu, Wuwei Yang, Mei Guo, Xiaoqing Liu, Naixiang Huang, Dingfeng Li, Zefei Jiang, Wenfeng Yang, Weijing Zhang, Hang Su, Zhiqing Liu, Tieqiang Liu, Dongmei Wang, Shan Huang, Bo Yao, Qiuhong Man, Lijuan Qiu, Xuedong Sun, Yuying Sun, Bing Liu

**Affiliations:** 1 Department of Hematology and Transplantation, Affiliated Hospital of the Academy of Military Medical Sciences, Dongdajie 8 Beijing, 100071 China; 2 Cancer Therapy Center, Affiliated Hospital of Academy of Military Medical Sciences, Dongdajie 8, Beijing, 100071 China

**Keywords:** Cytokine, Treg and NK, Cellular immunity, Immunotherapy, Immune modulation

## Abstract

Overinduced CD4^+^CD25^+high^ regulatory T cells (Treg) and downregulated NK cells contribute to tumor-relevant immune tolerance and interfere with tumor immunity. In this study, we aimed to design a novel strategy with cytokine combination to correct the dysregulated Treg and NK cells in malignant patients. Initially, a total of 58 healthy individuals and 561 malignant patients were analyzed for their cellular immunity by flow cytometry. The average percentages of CD4^+^CD25^+high^/lymphocyte were 1.30 ± 1.19 % ($\bar{x}$ ± SD) in normal adults and 3.274 ± 4.835 % in malignant patients (*p* < 0.001). The ratio of CD4^+^CD25^+high^ to CD4^+^ was 3.58 ± 3.19 % in normal adults and 6.01 ± 5.89 % to 13.50 ± 23.60 % in different kinds of malignancies (*p* < 0.001). Of normal adults, 15.52 % had >3 % Treg and 12.07 % had <10 % NK cells. In contrast, the Treg (>3 %) and NK (<10 %) percentages were 40.82 and 34.94 % in malignant patients, respectively. One hundred and ten patients received the immunomodulation therapy with IFN-α and/or IL-2. The overinduced Treg in 86.3 % and the reduced NK cells in 71.17 % of the patients were successfully modulated. In comparison, other lymphocyte subpopulations in most patients were much less affected by this treatment. No other treatment-relevant complications except slight pyrexia, fatigue, headache, and myalgia were observed. In conclusion, dysregulated Treg and/or NK cells were common in malignant patients. Different from any regimens ever reported, this strategy was simple and effective without severe complications and will become a basic regimen for other cancer therapies.

**Electronic supplementary material:**

The online version of this article (doi:10.1007/s00262-012-1297-2) contains supplementary material, which is available to authorized users.

## Introduction

Immune tolerance can be considered as another cause of cancer progression in malignant patients, and so far, clinical failures in cancer immunotherapy have largely been attributed to dysfunction of tumor immunity in most malignant patients. It has been well demonstrated that tumor-induced immune tolerance mostly arises from elevated immunosuppressive factors such as TGFβ [1], IL-10 [2], or PGE [3] and from tumor-induced modulatory cells with immunosuppressive properties such as those belonging to the subpopulation of T cells, dendritic cells (DCs), and the like [4, 5]. Particularly, the CD4^+^CD25^+high^ regulatory T cells (Treg) derived from naturally existing Treg cells or mostly converted from peripheral naive CD4^+^ T cells are considered to play a crucial role in tumor immunotolerance [6–8]. Moreover, the effect of natural killer (NK) cell dysfunction on tumor immunity has also been well investigated [9, 10].

Treg has a wide inhibitory effect on the immune system, that is, CD4^+^ T cells [11], CD8^+^ T cells [12], NK cells [13], B cells [14], natural killer T (NKT) cells [15], and dendritic cells [16, 17], and is highly induced in various types of tumors [18–21]. Several studies on animal models have proved that the tumor immunosurveillance can be augmented when CD4^+^CD25^+^ Treg is inhibited or depleted [22–24]. Therefore, multiple strategies have been designed to inhibit or deplete them to evoke tumor immunity, that is, CD25 monoantibody-toxin such as daclizumab, chemical anticancer drugs such as cyclophosphamide, or immunosuppressors such as fludarabine [25–27]. Nevertheless, the use of cyclophosphamide and fludarabine for the depletion/inhibition of Treg is either nonspecific or less efficient with chemical drug toxicities. The immunotoxin denileukin diftitox (Ontak) has been proved to be much reliable to selectively eliminate CD25-expressing Treg from PBMCs of malignant patients without toxicity. It is specific and effective but cannot be used repeatedly [23]. Moreover, the number of Treg cells after the depletion of CD25^+^ cells is generally restored over time, and the capacity to mount an antitumor response progressively diminishes [23, 25, 27]. Other studies have shown that depleting Treg cells also raises the possibility of autoimmunity [28].

NK cells play a crucial role in immunosurveillance [29]. It has been proved that inhibition of NK function contributes to poor tumor immunity [30, 31]. In addition, they are key effectors of antibody-dependent cell-mediated cytotoxicity (ADCC), being validated in the study of rituximab, a chimeric mouse–human antibody that recognizes the CD20 antigen expressed on mature B cells, and several other antibodies [32, 33]. These facts above suggest that either the number or the function of NK cells is important to tumor immunity. Several strategies have been investigated to enhance NK-cell responses to tumors experimentally or clinically, including the combined use of cytokines (such as interleukin-2 (IL-2)) and transfusion of in vitro expanded or activated autologous or allogeneic NK cells [34–36]. However, the result seems promising but still far from satisfactory.

Since the middle of 1990s, IFN-α and IL-2 have long been used separately or in combination in the treatment for malignancies, to evoke stronger antitumor immune response as immune modulators or to stimulate immune cells as growth factors, but the clinical antitumor outcomes were limited [37, 38]. Recently, in the application of IFN-α for the treatment of melanoma, we occasionally found that the upregulated CD4^+^CD25^+high^ cells in some patients simultaneously declined following the injections of IFN-α. Though the exact mechanism is still unknown, it is believed that it may come from the tendency of the modulatory activities of IFN-α and IL-2 toward different lymphocyte subsets. This new finding led us to design a new regimen to overcome tumor-induced upregulation of Treg or inhibition of NK for therapeutic purpose.

In the present study, we found that the dysregulated Treg (>3 %) and/or NK cells (<10 %) were common in malignant patients. By combining IFN-α and IL-2, the Treg and NK cells could be selectively and effectively modulated without severe complications, achieving the balance of the cellular immunity. The strategy is promising to become a basic treatment for other cancer therapies and adds new dimension to cancer immunotherapy.

## Patients and methods

### Patients and eligibility

A total of 58 healthy individuals and 561 various malignant patients (aged from 25 to 76, 360 males and 201 females) from the Cancer Therapy Center in our hospital were enrolled in this study. All patients were tumor bearing on physical examination or radiographic imaging during their visits to the hospital regardless of the cancer types and the treatments received in the clinic. The cellular immunity of the patients was continuously monitored for 2–23 months, with the minimum interval over 1 month.

### Analysis of cellular immunity by flow cytometry

Peripheral blood was analyzed by a 4-color flow cytometry (EPICS XL, Beckman Coulter Inc., USA). Fluorescein isothiocyanate (FITC)-, PE-Cy5-, PerCP-, allophycocyanin (APC)-, or PE-Texas Red (ECD)-conjugated antibodies against CD3, CD4, CD8, CD56, CD19, CD25, CD28, HLA-DR, CD45RA, and CD45RO were purchased from Beckman Coulter Inc. Cells were labeled according to the manufacturer’s protocols. The data were initially collected on day −3 to day 0 before treatment. The post-treatment data were collected immediately after treatment, mostly on the last day of a course. Only a few outpatients had the analysis on their convenience with days or weeks delay. The follow-up interval was generally recommended as 4–6 months.

### Regimens for modulation of the cellular immunity

Upon approval by the Ethics Committee of the Affiliated Hospital of the Academy of Military Medical Sciences, a total of 110 patients received the immune modulation therapy after giving informed consent. IFN-α-1b (Beijing Tri-Prime Gene Inc.) and IL-2 (Beijing Shuanglu Pharmaceutical Inc. China) were used. The doses were 300 MU of IFN-α and 200 MU of IL-2 for each injection. The protocols of the therapy were designed according to the status of CD4^+^CD25^+high^ and NK cells: (1) type 1 treatment: if CD4^+^CD25^+high^ cells exceeded 3 % of the lymphocyte population or accounted for 30 % of the CD4^+^ population, IFN-α-1b was used subcutaneously at every other day for 3 weeks for each course of treatment. In early stage of this study, it was given daily in some patients; (2) type 2 treatment: if NK cells were <10 % with CD4^+^CD25^+high^ <3 %, 1 day of IFN-α-1b was followed by 2 days of IL-2 for 3 weeks for each course of treatment; (3) type 3 treatment: if the patients had >3 % CD4^+^CD25^+high^ and <10 % NK cells, IFN-α-1b was used daily until CD4^+^CD25^+high^ cells were dropped to near 3 %, then 1 day of IFN-α-1b followed by 2 days of IL-2 for 3 weeks. In addition, the following prerequisites were needed for the patients to receive the therapy: the absolute neutrophil count >0.3 × 10^9^/L and platelet count >70 × 10^9^/L, normal liver and renal function, at least 2 weeks after chemotherapy or operation, after wound healing, without gastrointestinal tract bleeding, and without a history of allergy to cytokines. If overinduced Treg and downregulated NK were not reversed (Treg/lymphocyte <3 %, NK/lymphocyte >10 %), another course was given.

### Statistical analysis

SASS 9.0 software was used for all statistical analyses. The significance of the results was determined using the rank sum test. Statistical significance was defined as *p* < 0.001.

## Results

### CD4^+^CD25^+high^ T cells in normal adults and various malignant patients

**Fig. 1:**
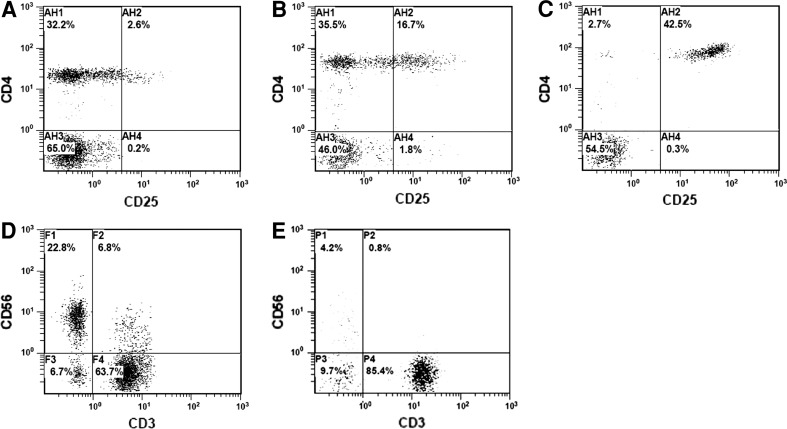
Treg and NK cells in normal adults and malignant patients. Flow cytometry analysis shows the Treg (CD4^+^CD25^+high^) in representatives of normal adults (**a**) and malignant patients (**b**, **c**), and the NK cells (CD3^−^CD56^+^) in representatives of normal adults (**d**) and malignant patients (**e**)

CD4^+^CD25^+high^ T cells were observed in both the lymphocyte and the CD4^+^ populations. Among 58 normal adults, the average proportion of CD4^+^CD25^+high^ cells was 1.30 ± 1.19 % ($\bar{x}$ ± SD) (range 0.1–4.3 %, a representative one shown in Fig. 1a) in the lymphocyte population and 0.23–10.73 % in the CD4^+^ population. Nine of them (15.52 %, 9/58) had their Treg/lymphocyte over 3 %. Four (6.9 %, 4/58) had their Treg/CD4^+^ over 10 %, and one (1.72 %, 1/58) over 15 %. The average proportion of NK was 20.44 ± 10.09 % (range 5.8–67.6 %, a representative one shown in Fig. 1d). Seven (12.07 %, 7/58) had their NK/lymphocyte reduced to less than 10 %. Only one (1.72 %, 1/58) had both elevated Treg (Treg/lymphocyte >3 %) and downregulated NK (<10 %) (Table 1).

**Table 1:** Treg and NK cells in different cancers

Individuals	CD4/CD8	Treg/Lym (%)	Treg/CD4^+^ (%)	NK/Lym (%)
Normal adults (*n* = 58)	1.30 ± 0.58	1.30 ± 1.19	3.58 ± 3.19	20.40 ± 10.09
Lung cancer (*n* = 126)	1.63 ± 1.21	2.94 ± 2.00	9.50 ± 7.42	16.24 ± 9.70
Breast cancer (*n* = 52)	1.45 ± 0.65	3.42 ± 7.78	10.35 ± 23.37	15.55 ± 7.66
Ovarian cancer (*n* = 23)	1.65 ± 1.19	4.35 ± 4.46	10.99 ± 9.36	17.50 ± 12.34
Gastrointestinal cancer (*n* = 88)	1.52 ± 0.86	3.00 ± 1.97	9.81 ± 13.11	17.49 ± 8.90
Renal carcinoma (*n* = 45)	1.68 ± 0.88	5.70 ± 10.74	13.50 ± 23.60	12.11 ± 8.68
Melanoma (*n* = 14)	1.34 ± 1.18	2.56 ± 2.96	8.62 ± 10.58	18.67 ± 10.05
Sarcoma (n = 41)	1.97 ± 1.26	2.21 ± 2.10	6.01 ± 5.89	9.98 ± 5.80
Intracranial cancer (*n* = 10)	2.10 ± 0.84	2.77 ± 1.57	6.77 ± 5.05	10.46 ± 5.49
Pancreatic cancer (*n* = 19)	1.58 ± 0.81	3.25 ± 2.89	8.62 ± 7.93	13.09 ± 6.70
Liver cancer (*n* = 31)	1.29 ± 1.01	3.71 ± 6.29	12.48 ± 17.89	15.72 ± 9.54
Other cancers (*n* = 112)	1.45 ± 0.86	3.10 ± 4.14	9.56 ± 13.11	14.22 ± 9.77

The value is expressed as mean ± SD. *Lym* lymphocytes

Among the malignant patients, the average proportion of CD4^+^CD25^+high^ cells was 3.27 ± 4.86 % ($\bar{x}$ ± SD), ranging from 0.1 to 54.1 % of their lymphocyte population (two representatives shown in Fig. 1b, c) and 0.22–177.18 % of their CD4^+^ population, significantly higher than that in normal adults (*p* < 0.001); 229 patients (40.82 %, 229/561) had their Treg/lymphocyte over 3 %, and 183 cases (32.62 %, 183/561) had their Treg/CD4^+^ over 10 % and 96 cases (17.11 %, 96/561) over 15 %. Particularly, the percentages of patients with Treg/CD4^+^ > 10 % and >15 % were 2.3–5.3 and 5.8–14.5 times over those of the normal adults, respectively (see Table 2). The average proportion of NK cells was 14.93 ± 9.31 % ($\bar{x}$ ± SD, ranging from 0.1 to 54.5 %, a representative one shown in Fig. 1e) in malignant patients, significantly lower than that of normal adults (*p* < 0.001). One hundred and ninety-six cases (34.94 %, 196/561) had reduced NK cells to less than 10 % of their lymphocyte population. Notably, 75 cases (13.37 %, 75/561) had both overinduced Treg (Treg/lymphocyte > 3 %) and reduced NK (<10 %). Regarding the Treg ratio, there was a significant difference between the normal adults and the patients with lung cancer (*p* < 0.001), gastrointestinal cancer (*p* < 0.001), intracranial cancer (*p* < 0.001), ovarian cancer (*p* < 0.001), pancreatic cancer (*p* < 0.001), renal cancer (*p* < 0.001), and other malignancies (*p* < 0.001), respectively. With respect to the NK ratio, there was a significant difference between the normal adults and the patients with intracranial cancer (*p* < 0.001), ovarian cancer (*p* < 0.001), renal cancer (*p* < 0.001), breast cancer (*p* < 0.001), sarcoma (*p* < 0.001), and the group of other malignancies (*p* < 0.001), respectively.

**Table 2:** Proportion of patients with overinduced Treg and downregulated NK cells in different cancers

Individuals	CD4/CD8 (<1) (%)	NK/Lym (<10) (%)	Treg/Lym (>3) (%)	Treg/CD4^+^ (>10) (%)	Treg/CD4^+^ (>15) (%)
Normal adults (*n* = 58)	35.59	12.07	15.52	6.90	1.72
Lung cancer (*n* = 126)	35.62	28.09	41.10	32.88	17.13
Breast cancer (*n* = 52)	24.57	19.30	26.32	24.57	10.53
Ovarian cancer (*n* = 23)	39.13	34.78	47.83	30.43	17.39
Gastrointestinal cancer (*n* = 88)	34.09	23.86	36.35	30.68	15.91
Renal carcinoma (*n* = 45)	14.59	43.75	41.67	33.34	24.44
Melanoma (*n* = 14)	44.45	16.67	22.23	16.67	11.12
Sarcoma (*n* = 41)	22.73	52.28	24.39	20.46	11.37
Intracranial cancer (*n* = 10)	0	40.00	40.00	20.00	10.00
Pancreatic cancer (*n* = 19)	21.05	26.32	42.11	36.84	15.79
Liver cancer (*n* = 31)	54.58	22.58	35.48	32.26	12.9
Other cancers (*n* = 112)	33.05	45.22	36.53	27.83	14.79

*Lym* lymphocytes

### Selective modulation of CD4^+^CD25^+high^ and NK populations in patients with different cancers

**Fig. 2:**
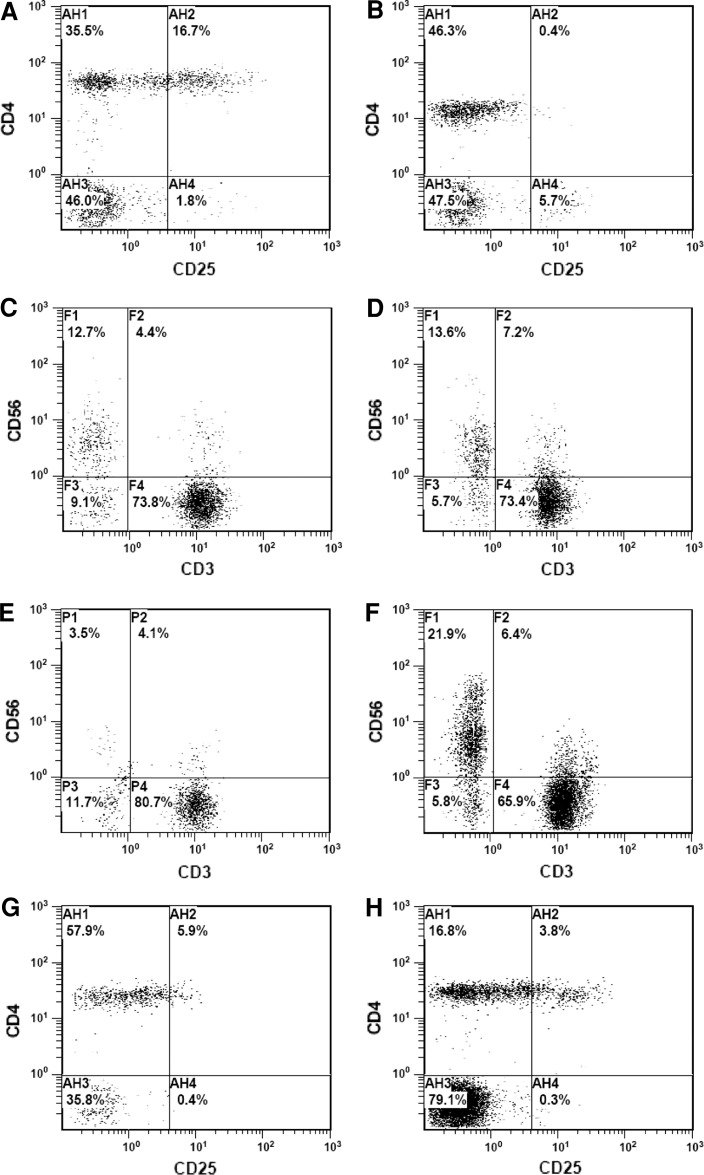
Modulation of dysregulated Treg and NK cells in malignant patients. After type 1 treatment, the overinduced Treg (**a**, CD4^+^CD25^+high^) in a representative malignant patient could be modulated to 0.4 % (**b**), and the NK cells remained normal (**c**, **d**). After type 2 treatment, the downregulated NK (**e**, CD3^−^CD56^+^) in a representative malignant patient could be elevated to 21.9 % (**f**), and the Treg remained normal (**g**, **h**)

Based on the above results, 3 % of CD4^+^CD25^+high^ cells and 10 % of NK cells in the lymphocyte population were determined as the reference levels. The patients who had >3 % Treg and/or <10 % NK cells were considered to have imbalance in their cellular immunity (overinduced immunosuppression) and were chosen for immunomodulatory therapy. A total of 110 patients received the immune modulation. After treatment, the Treg/lymphocyte ratio was successfully downregulated to <3 % in 86.3 % (63/73) of the patients (data from a representative patient shown in Fig. 2a–d) and the NK/lymphocyte ratio to >10 % in 71.19 % (42/59) of the patients (data from a representative patient shown in Fig. 2e–h). Most therapies finished within 3 weeks, and 17 patients finished in 1–3 months (mostly within 2 months, until Treg/lymphocyte <3 % and NK/lymphocyte >10 %). Owing to the unavailability of the methodology in our laboratory during early years, the expression of Foxp3 in the CD4^+^CD25^+high^ T-cell population was not analyzed initially in this study. Regarding the Foxp3-expressing CD4^+^CD25^+high^ T cells, we further analyzed 10 samples of normal adults from the blood bank in our hospital and 6 samples of malignant patients with routine immunomodulatory treatment (Supplementary Figs. 5, 6). All of the normal adults and five patients had low levels of CD4^+^CD25^+high^ as well as CD4^+^CD25^+high^ Foxp3^+^ T cells initially. One patient (#1) had overinduced CD4^+^CD25^+high^ and CD4^+^CD25^+high^Foxp3^+^ T cells. After treatment, the overinduced CD4^+^CD25^+high^ and the CD4^+^CD25^+high^Foxp3^+^ T cells were effectively downmodulated to low level, while those in the five other patients were maintained at low levels. Currently, we have initiated a large cohort multi-center observation regarding the modulatory efficacy of this novel strategy on the CD4^+^CD25^+high^Foxp3^+^ Tregs in malignancies.

**Fig. 3:**
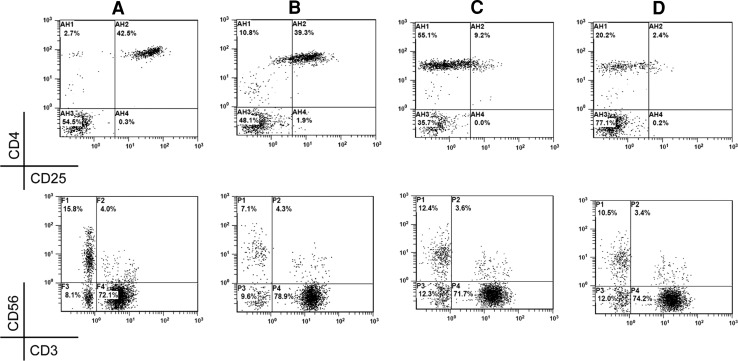
Correction of fluctuated Treg and NK cells by multiple types of treatment. After type 1 treatment, the NK cells (CD3^−^CD56^+^, *lower row*) in a malignant patient with overinduced Treg (CD4^+^CD25^+high^, upper row) (**a**) were downregulated to 7.1 % (**b**). Upon subsequent two courses of type 3 treatment, the NK population became >10 % (**c**). After another type 1 treatment, the Treg was <3 % with >10 % NK population (**d**)

**Fig. 4:**
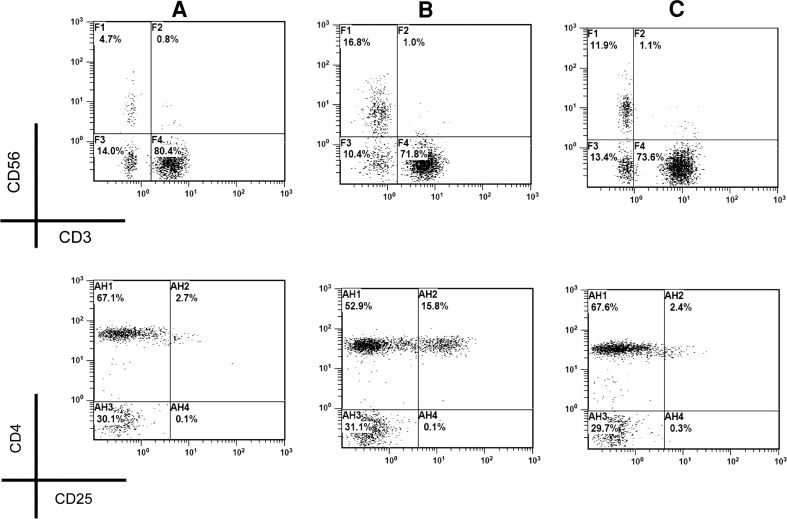
Correction of fluctuated NK and Treg cells by multiple types of treatment. After type 2 treatment, the Treg (CD4^+^CD25^+high^, *lower row*) in a malignant patient with downregulated NK population (CD3^−^CD56^+^, *upper row*) (**a**) was overinduced to 15.8 % (**b**). Upon subsequent several courses of type 1 treatment, the Treg population became <3 % with >10 % NK (**c**)

Meanwhile, after IL-2 treatment, the Treg/lymphocyte ratio was still >3 % in 47 patients, but 34 (72.34 %) of them were successfully reversed to <3 % by a following course of type 1 treatment and 13 missed the follow-up. After the use of IFN-α-1b, the NK population was still <10 % in 24 patients. Following a course of type 2 or type 3 treatments, the ratio was successfully upregulated in 12 patients (50 %), and the other 12 missed the follow-up. Notably, some patients had frequent fluctuation of Treg and NK during the treatment. Therefore, consecutive monitoring and repeated modulation were required. As shown in Fig. 3, after two courses of type 1 treatment against overinduced Treg (42.5 %), the NK population dropped to 7.1 %. Then, two courses of type 3 treatment were given, and the Treg and NK reached 9.2 and 12.4 %, respectively. Thereafter, another type 1 treatment was given, and the final Treg and NK population were 2.4 and 10.5 %, respectively. Figure 4 shows another patient with primary downregulated NK (4.7 %) and normal Treg (2.7 %). After two courses of type 2 treatment, the NK population became 16.8 % but the Treg increased to 15.8 %. After several courses of type 3 and type 1 treatments, the NK and Treg were finally modulated to 11.9 and 2.4 %, respectively.

The influence of the therapy on other lymphocytes, such as CD3^+^, CD3^+^CD4^+^, CD3^+^CD8^+^, CD3^−^CD19^+^ subpopulations, was separately analyzed (Supplemental Table 2). When dysregulated Treg and NK cells were successfully corrected, the CD3^+^, CD3^+^CD4^+^, CD3^+^CD8^+^, and CD3^−^CD19^+^ subpopulations were slightly affected in both up and down directions in most cases. In comparison, more patients had CD3^+^ and CD3^+^CD4^+^ cells downmodulated and CD3^+^CD8^+^ cells upmodulated. To minimize the interference of the Treg and NK modulations on other lymphocyte populations, the dose was limited to 200 MU of IL-2 and/or 300 MU of IFN-α-1b per day, the length of each course limited to less than 3 weeks, and the interval of treatment courses or follow-up limited to 4–6 months or longer.

Of all the patients received the treatment, no other treatment-relevant complications except pyrexia, fatigue, headache, and myalgia were observed. No anaphylactic or autoimmune disease was observed in any of the patients.

## Discussion

In this study, to overcome the tumor-induced immunotolerance, a simple and safe strategy with IFN-α and/or IL-2 was used to selectively and efficiently downregulate Treg and upregulate NK cells in malignant patients. The result showed that the overinduced Treg in 86.3 % and the reduced NK cells in 71.17 % of the patients were successfully modulated by one course of treatment.

As all the patients enrolled had detectable tumor burden on imaging examination, the changes in Treg and NK cells may reflect in vivo perturbation of the cancer-relevant factors on cellular immunity. The results revealed that Treg cells were highly induced in tumor-bearing patients, which confirmed to the reports from the literatures [18–21]. Interestingly, the significant difference (*p* < 0.001) in Treg ratio was observed between the normal adults and the patients with lung cancer, gastrointestinal cancer, intracranial cancer, ovarian cancer, pancreatic cancer, renal cancer, or group of other malignancies. For each kind of cancer, a significant proportion of the patients had their Treg/lymphocyte > 3 % and Treg/CD4^+^ > 10 % or >15 %, respectively. These results indicated that Treg-mediated immunosuppression seemed to be a general mechanism of tumor immunotolerance, but depended on random/ongoing immunosuppressive induction of malignancies. In addition, the NK proportion in malignant patients was also frequently downregulated. As documented previously, Treg can inhibit NK in both direct and indirect ways [37, 39, 40]. In this study, 13.37 % (75/561) of the patients had both overinduced Treg and downregulated NK cells. In the 196 patients with primarily downregulated NK cells (<10 %), only 38.27 % (75/196) had overinduced Treg. The result indicated that there was no significant correlation between the highly induced Treg and downregulated NK cells. The other lymphocytes, such as CD3^+^, CD3^+^CD4^+^, CD3^+^CD8^+^, CD3^−^CD19^+^ subpopulations, were slightly affected by the immunomodulatory treatment in most cases. The influence and its importance were much less in comparison with the correction of dysregulated Treg and NK cells. Moreover, as each course of the treatment was limited to about 3 weeks and these lymphocyte populations could be automatically remodulated by the immune system itself in weeks, we did not observe any impact of them to the immune function.

To date, many strategies have been employed clinically to induce tumor immunity, such as cytokines, vaccines, in vitro activated immune cells, and even donor lymphocyte infusion (DLI) [38, 41–43]. Despite the documented effects, the clinical outcome is far from satisfactory. It is believed that tumor-induced immunotolerance in vivo is one of the main obstacles that block or interfere with the antitumor function of the immune system or the adoptive immune cells [39]. Therefore, it is critical for cancer treatment to effectively reverse the immunotolerance prior to or during any clinical antitumor therapy. The selective downregulation of overinduced Treg described here may contribute to cancer treatment based on at least the partial reduction of Treg-related immunotolerance and the improvement of the immunoenvironment of malignant patients. Concerning the successful modulation of NK cells in vivo, the approach may also be significant for NK-mediated cancer therapy, apart from other conventional regimens (i.e., transfusion of autologous or allogeneic NK cells and even administration of IL-2) [31, 34–36]. Moreover, IFN-α and/or IL-2 has been used in the treatment for malignancies for decades via modulating immunity. Several systematic and long-term studies have proved their effectiveness and safety, including the combined use of them for the treatment of advanced renal cell carcinoma [37–41]. In this study, the administration of cytokines is mainly for overcoming tumor-induced immunotolerance and maintaining the balance of cellular immunity. Importantly, different from other strategies for Treg inhibition/depletion or NK activation, this strategy mimics the physical immunoregulation by cytokines in vivo, and can be used timely and repeatedly, based on the status of cellular immunity and the balance between different lymphocyte subsets.

Furthermore, by monitoring the shifting of Treg and NK as well as the balance of the cellular immunity during disease progression, clinical treatment, and the immunomodulation itself, we observed frequent up- or downregulation of the lymphocyte subsets, including the overinduced Treg and downregulated NK at an interval of weeks to months. It may be caused by factors from tumors and administration of IL-2 (overinducing Treg) or IFN-α (downregulating NK) [18–21]. It is believed that various factors from diseases and agents/drugs may induce such shifting of the cellular immunity (e.g., from their natural immunoregulatory activities). The immunoregulatory activity of cytokines is the basis of our novel strategy, which may also cause new imbalance of cellular immunity. Such new imbalance may have the same impact on tumor immunity from cancers and should receive more attention when they are used.

To maintain tumor immunity and prevent the propagation of tumor immunotolerance in malignant patients, continuously monitoring and timely correcting the imbalance of the cellular immunity (overinduced Treg, downregulated NK, and the like) is needed during cancer treatment. This new treatment will provide optimal option for this purpose. Interestingly, as IFN-α can downregulate Treg, it can also prevent the induction of Treg by IL-2, calling for a much delicate design of the immunomodulation therapy and more modulators (cytokines or others). The outcome of the modulation can be further improved if more cytokines become clinically available.

With respect to the side effects of IFN-α and IL-2, it has been reported that pyrexia, fatigue, headache, and myalgia were generally observed, while severe complications such as hematopoietic inhibition or hepatic decompensation were occasionally seen in long-term use of them [42, 43]. In this study, as short-term administration and limited dose of IFN-α and IL-2 were employed, we observed no more than slight pyrexia, fatigue, headache, myalgia in some patients. Importantly, the signs directly correlated with autoimmune reaction or aggravation of pre-existed pathological phenomenon were not observed.

In conclusion, we reported a novel strategy using cytokines to selectively and effectively modulate Treg and NK cells and as a result, maintained the balance of the cellular immunity in malignant patients. The strategy itself is a kind of immunotherapy and can become a supplementary treatment for other kinds of cancer therapies. Further investigation will focus on optimization of this so called in vivo immunoediting of the cellular immunity strategy by employing more cytokines and also on its antitumor effectiveness, especially when it is employed in combination with other kinds of therapies.

## Electronic supplementary material

Below is the link to the electronic supplementary material.

Supplementary material 1 (DOC 3268 kb)
