# Supplementary material for: Effective modulation of CD4+CD25+high regulatory T and NK cells in malignant patients by combination of interferon-α and interleukin-2
Source: Cancer Immunol Immunother. 2012 Jun 22;61(12):2357–66. doi: 10.1007/s00262-012-1297-2 (PMC3506201; doi:10.1007/s00262-012-1297-2)
Supplement: Supplementary file 1 — Supplementary material 1 (DOC 3268 kb) [file 262_2012_1297_MOESM1_ESM.doc]

**Supplementary Data:**

**Supplementary Table 1** Treg and NK modulation by cytokines in malignant patients

|  | Treg downmodulated only | NK upregulated only | Both Treg downmodulated and NK upregulated | |
| --- | --- | --- | --- | --- |
|  |  |  | Treg | NK |
| cases | 22 | 25 | 33 | 33 |
| Modulated range in the lymphocyte population (mean ± SD) | 1.6-16.2% (5.25±3.53%) | 3.1-21.7% (9.7±5.27%) | 1.4-57.1% (5.79±9.5%) | 2.2-36.2% (10.37±8.07%) |
| Modulated range over the original levels (mean ± SD) | 38.1-99.3% (79.4±16.7%) | 32.3-715.4% (197.03±175.98%) | 32.2-96.8% (69.04±18.56%) | 17.1-861.2% (154.37±194.75%) |

**Supplementary Table 2** Influence of Treg and NK modulation on other lymphocyte subsets

|  |  |  | Treg down-modulation only | NK up-modulation only | Bothe Treg down-modulation and NK up-modulation |
| --- | --- | --- | --- | --- | --- |
| CD3 | Down-regulated | Cases (total) | 14 (22) | 21 (25) | 31 (33) |
|  |  | Modulated range in the lymphocyte population (mean ± SD) | 0.5-7.4% (3.87±2.76%) | 0.1-17.4% (8.31±4.89%) | 0.1-27.6% (9.71±7.51%) |
|  |  | Modulated range over the original levels (mean ± SD) | 0.8-11% (mean:5.61±3.99%) | 0.1-23.8% (10.07±6.68%) | 0.1-32.5% (12.32±8.89%) |
|  | Up-regulated | Cases (total) | 8 (22) | 4 (25) | 2 (33) |
|  |  | Modulated range in the lymphocyte population (mean ± SD) | 0.2-5.6% (2.44±2.06%) | 0.4-10.5% (4.78±4.35%) | 12.6-17.2% (14.9±3.25%) |
|  |  | Modulated range over the original levels (mean ± SD) | 0.3-11.3% (4.0±3.82%) | 0.5-15.6% (6.93±6.62%) | 22.0-48.7% (35.35±18.87%) |
| CD4 | Down-regulated | Cases (total) | 14 (22) | 17 (25) | 29 (33) |
|  |  | Modulated range in the lymphocyte population (mean ± SD) | 0.5-21.3% (8.31±5.67%) | 0.3-36.3% (9.94±10.22%) | 1.7-23.7% (11.24±6.26%) |
|  |  | Modulated range over the original levels (mean ± SD) | 0.7-48.9% (23.26±15.45%) | 0.9-65.6% (21.31±19.01%) | 4.1-47.8% (25.74±12.46%) |
|  | Up-regulated | Cases (total) | 8 (22) | 8 (25) | 4 (33) |
|  |  | Modulated range in the lymphocyte population (mean ± SD) | 0.7-13.5% (4.24±4.13%) | 0.5-13.5% (4.66±4.77%) | 0.1-29.3% (9.78±13.62%) |
|  |  | Modulated range over the original levels (mean ± SD) | 2.7-56.7% (14.46±17.9%) | 0.9-48.2% (16.66±19.17%) | 0.4-174.4% (54.62±81.98%) |
| CD8 | Down-regulated | Cases (total) | 11 (22) | 12 (25) | 10 (33) |
|  |  | Modulated range in the lymphocyte population (mean ± SD) | 0.1-21.8% (4.53±6.02%) | 2.0-16.1% (6.45±5.83%) | 0.1-13.3% (5.69±4.75%) |
|  |  | Modulated range over the original levels (mean ± SD) | 0.3-77.6% (16.66±21.66%) | 6.1-48.1% (19.28±13.02%) | 0.6-73.9% (21.26±21.09%) |
|  | Up-regulated | Cases (total) | 11 (22) | 13 (25) | 23 (33) |
|  |  | Modulated range in the lymphocyte population (mean ± SD) | 1.1-17.6% (7.51±5.94%) | 0.2-24.2% (6.18±7.02%) | 0.1-16.2% (5.35±4.57%) |
|  |  | Modulated range over the original levels (mean ± SD) | 3.5-84.2% (27.55±29.15%) | 0.8-101.2% (27.75±32.27%) | 0.3-117.6% (28.08±27.84%) |
| CD19 | Down-regulated | Cases (total) | 7 (22) | 14 (25) | 18 (33) |
|  |  | Modulated range in the lymphocyte population (mean ± SD) | 0.7-7.3% (3.53±2.31%) | 0.4-13.2% (4.87±3.32%) | 0.1-28.3% (5.78±7.49%) |
|  |  | Modulated range over the original levels (mean ± SD) | 28.0-82.1% (60.81±18.51%) | 22.2-94.7% (60.27±22.65%) | 1.1-72.2% (44.38±23.47%) |
|  | Up-regulated | Cases (total) | 15 (22) | 11 (25) | 25 (33) |
|  |  | Modulated range in the lymphocyte population (mean ± SD) | 0.1-10.2% (3.74±2.84%) | 0.3-15.2% (4.49±4.57%) | 0.1-21.7% (6.21±5.75%) |
|  |  | Modulated range over the original levels (mean ± SD) | 1.3-273.3% (107.99±84.44%) | 22.0-2171.4% (387.57±640.06%) | 1.5-687.5% (215.49±267.18%) |

Notes: When Treg or/and NK were modulated as in Table 1A, the other lymphocyte subsets such as CD3^+^, CD4^+^, CD8^+^ ,CD19^+^ cells were influenced simultaneously as in Table 2. Range of lymphocyte subsets: real percentage of lymphocyte subsets in blood modulated. Range modulated: percentage of lymphocyte subsets over their original level after Treg or/and NK modulation.

**Supplementary Table 3** Modulation of Treg and NK in different malignancies

|  | | Lung cancer | Gastrointestinal cancer | Breast cancer | Liver cancer | Pancreatic cancer | Sarcoma | Renal carcinoma | Melanoma | Others |
| --- | --- | --- | --- | --- | --- | --- | --- | --- | --- | --- |
| Total case number | | 33 | 20 | 9 | 7 | 7 | 7 | 5 | 5 | 17 |
| Treg down-modulation only | Cases of successful Treg-downregulation (total number) | 15 (16) | 8 (9) | 2 (2) | 2 (2) | 3 (3) | 1 (1) | 2 (2) | 1 (1) | 9 (11) |
|  | Treg average pre-treatment (mean ± SD) | 5.38%±2.2% | 5.66%±0.95% | 7.30%±3.54% | 5.15%±2.62% | 4.67%±2.29% | 4% | 5.15%±0.07% | 13.20% | 8.35%±4.35% |
|  | Treg average post-treatment (mean ± SD) | 1.91%±0.93% | 1.79%±1.14% | 1.40%±0.57% | 1.35%±0.21% | 1.47%±0.06% | 1.50% | 0.25%±0.07% | 0.10% | 2.25%±2.15% |
| NK up-modulation only | Cases of successful NK-upregulation (total number) | 6 (6) | 4 (4) | 3 (3) | 3 (3) | 3 (3) | 4 (4) | 2 (2) | 1 (1) | 2 (2) |
|  | NK average pre-treatment (mean ± SD) | 7.15%±2.69% | 5.4%±2.18% | 7.40%±2.69% | 5.77%±4.19% | 4.47%±3.16% | 7.50%±2.77% | 7.75%±0.78% | 2.60% | 8.20%±1.97% |
|  | NK average post-treatment (mean ± SD) | 18.33%±9.78% | 14.85%±3.87% | 14.30%±6.15% | 16.90%±3.22% | 12.33%±3.87% | 16.38%±3.48% | 18.45%±8.98% | 21.20% | 15.45%±2.76% |
| Both Treg down-modulation and NK up-modulation | Cases of successful Treg-downregulation (total number) | 7 (11) | 2 (7) | 3 (4) | 1 (2) | 1 (1) | 2 (2) | 1 (1) | 2 (3) | 2 (4) |
|  | Treg average pre-treatment (mean ± SD) | 6.75%±2.02% | 5.86%±2.41% | 18.83%±26.81% | 4.95%±1.77% | 8.50% | 4.95%±1.91% | 13.50% | 6.13%±3.29% | 5.08%±0.92% |
|  | Treg average post-treatment (mean ± SD) | 2.76%±1.74% | 3.63%±1.83% | 2.20%±1.57% | 2.00%±2.12% | 1.40% | 1.60%±0.14% | 2.50% | 3.06%±1.98% | 12.42%±19.94% |
|  | Cases of successful NK-upregulation (total number) | 8 (11) | 7 (7) | 4 (4) | 1 (2) | 1 (1) | 2 (2) | 1 (1) | 2 (3) | 4 (4) |
|  | NK average pre-treatment (mean ± SD) | 6.66%±2.35% | 8.2%±2.1% | 6.10%±2.26% | 3.35%±0.71% | 2.60% | 8.70%±0.71% | 4.60% | 4.5%±2.69% | 8.05%±1.14% |
|  | NK average post-treatment (mean ± SD) | 13.15%±5.62% | 16.59%±3.65% | 15.70%±3.89% | 7.75%±5.30% | 11.50% | 14.35%±1.20% | 13.90% | 10.47%±5.55% | 31.00%±14.50% |

**Supplementary Figure 1**


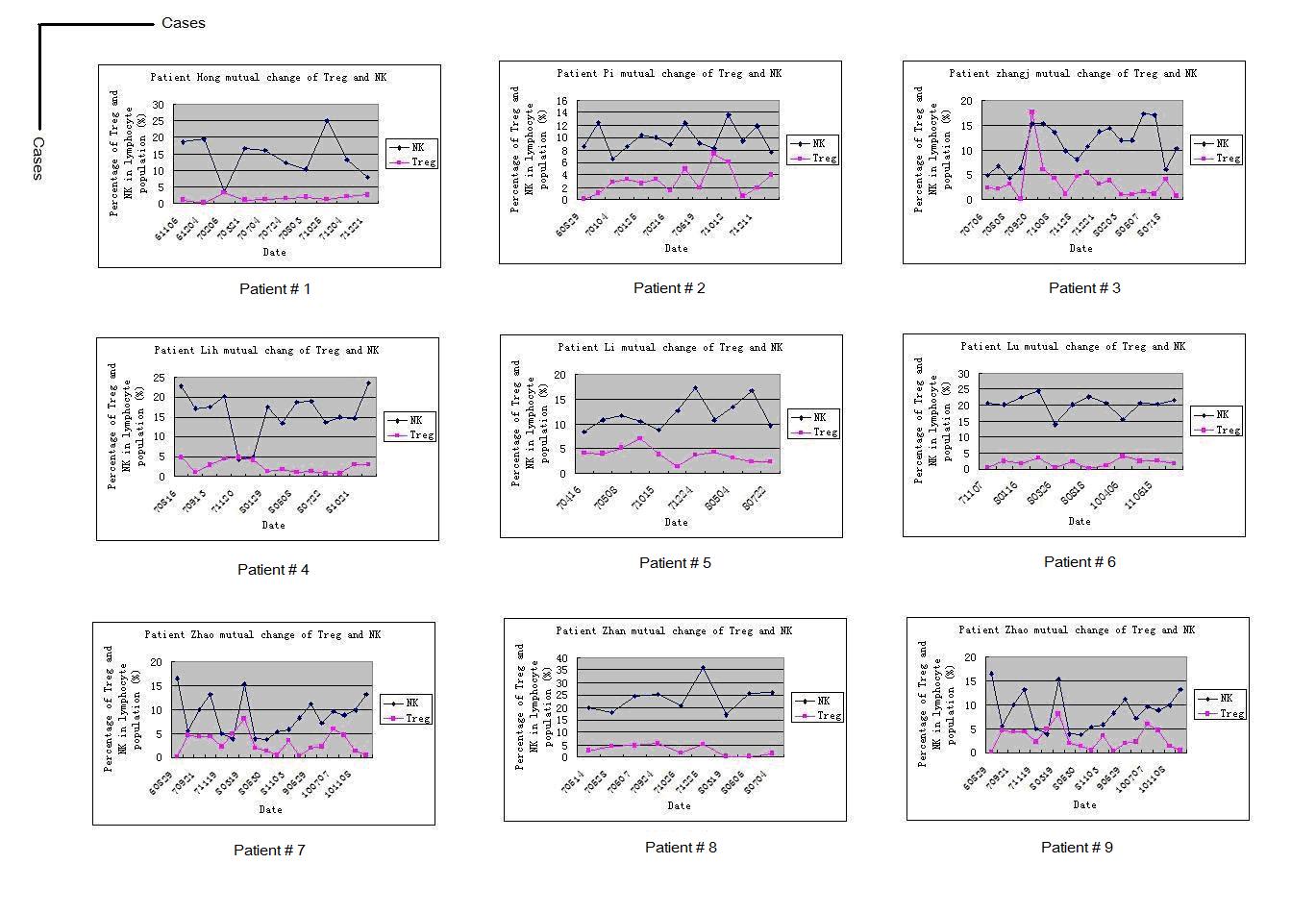


**Supplementary Figure 1** Examples of mutual changes of Treg and NK in the group of patients with both downmodulated Treg and upmodulated NK and the patients receiving long-term monitoring and immunomodulation therapy. Each pair of points representing Treg and NK at an individual date in the figures represent a measurement of the cellular immunity and maybe a following immunomodulatory treatment accordingly.

**Supplementary Figure 2**


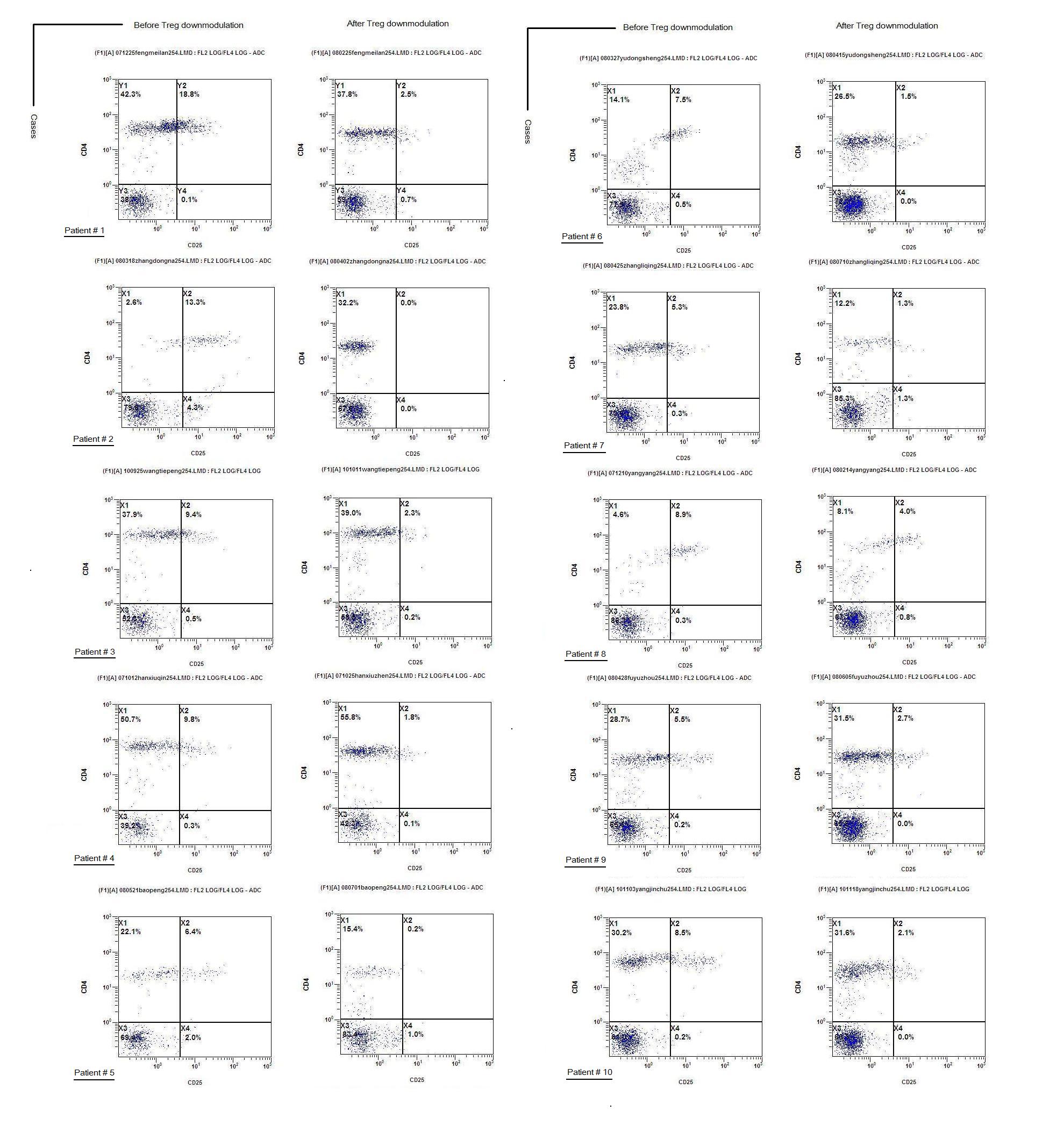


**Supplementary Figure 2** Histogram data of 10 representatives in the group of Treg down-modulation only.

**Supplementary Figure 3**

**
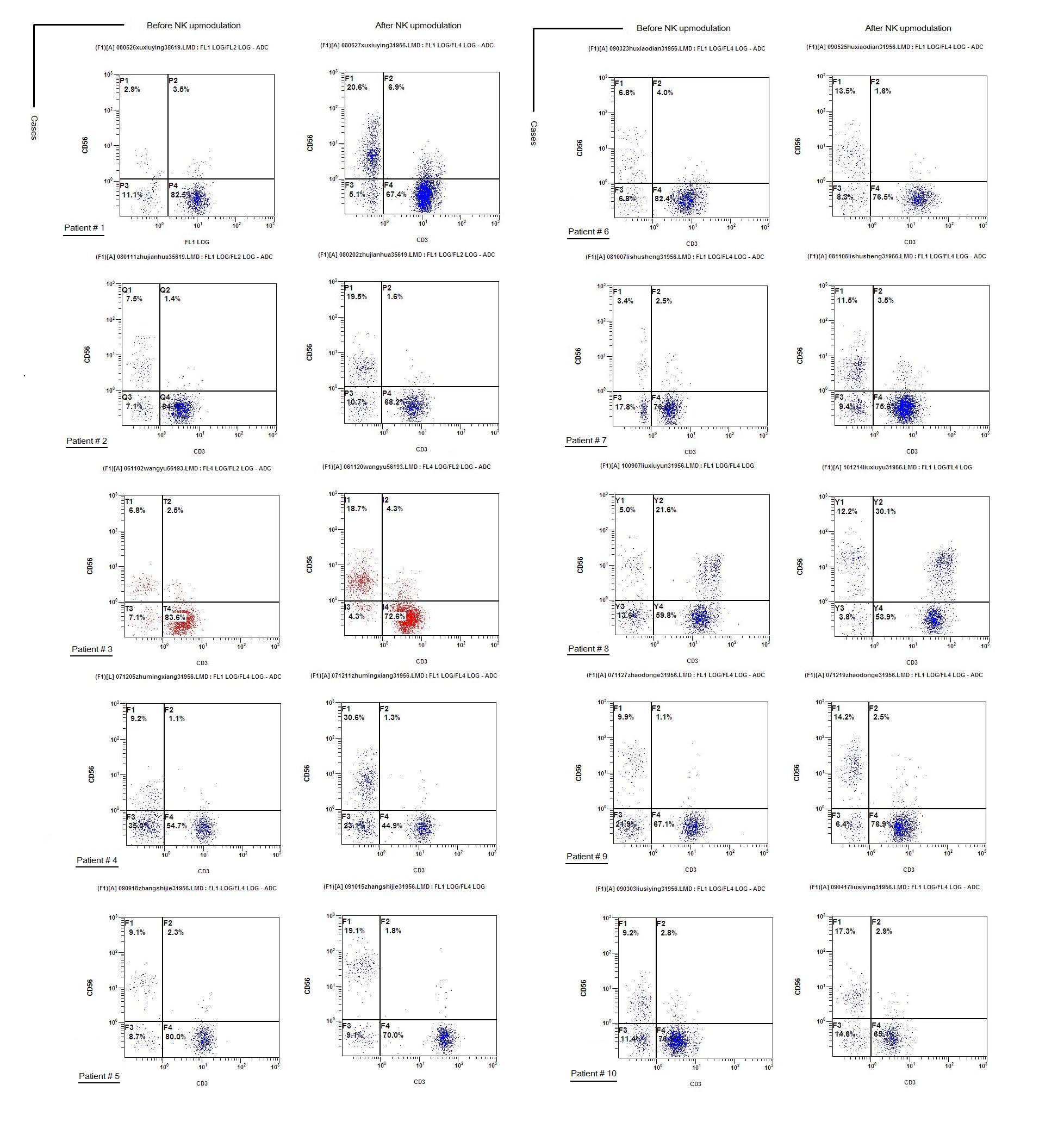
**

**Supplementary Figure 3** Histogram data of 10 representatives in the group of NK up-modulation only.

**Supplementary Figure 4**


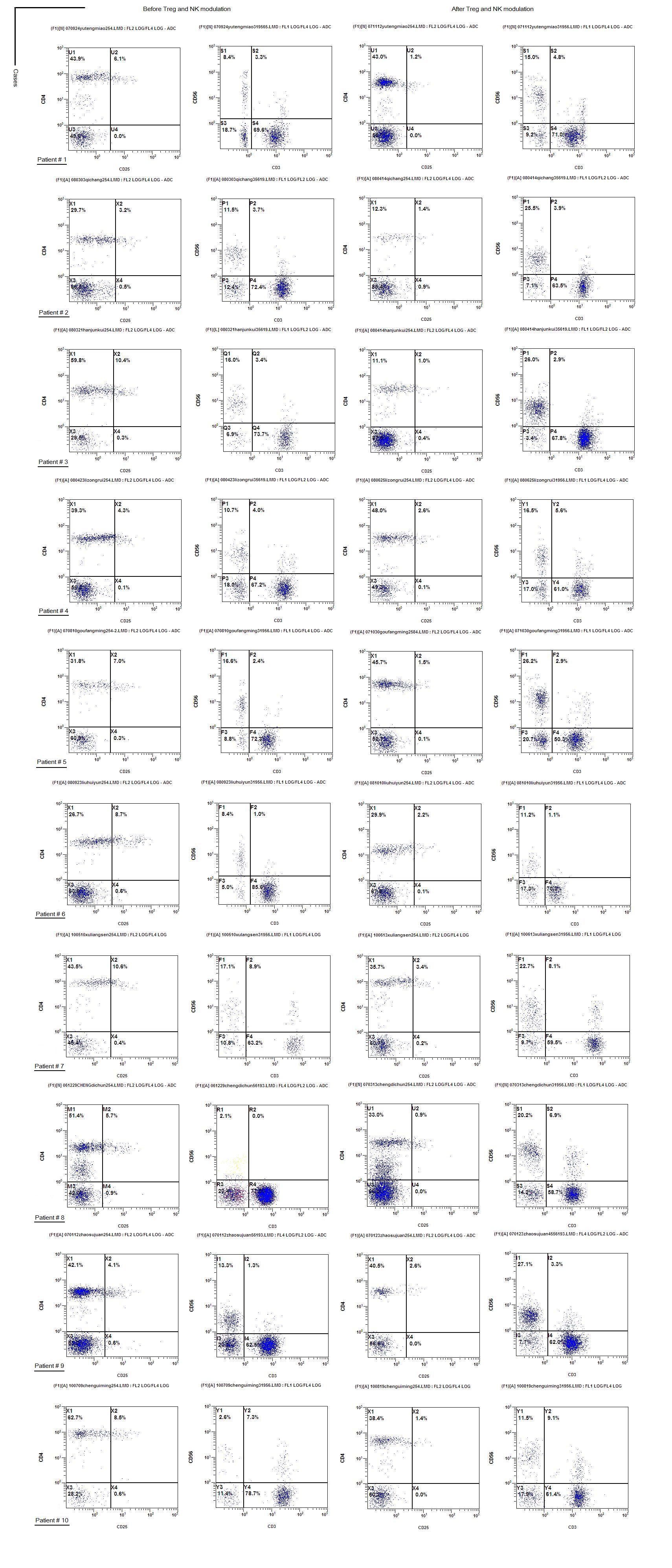


**Supplementary Figure 4** Histogram data of 10 representatives in the group of patients with both Treg down-modulation and NK up-modulation.

**Supplementary Figure 5**


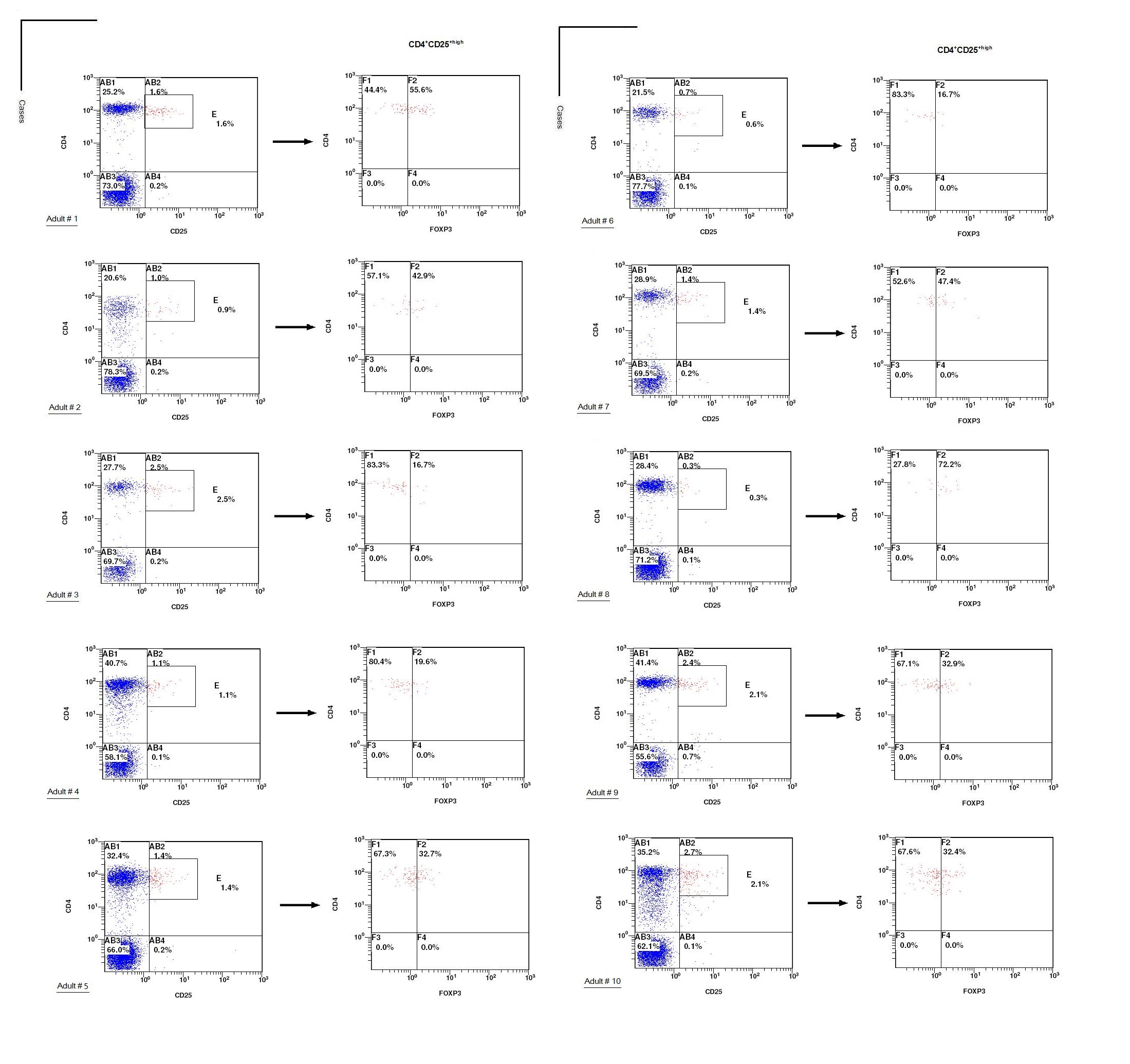


**Supplementary Figure 5** Histogram data of 10 normal adults who had low levels of CD4^+^CD25^+high^ T cells as well as CD4^+^CD25^+high^Foxp3^+^ T cells.

**Supplementary Figure 6**

**
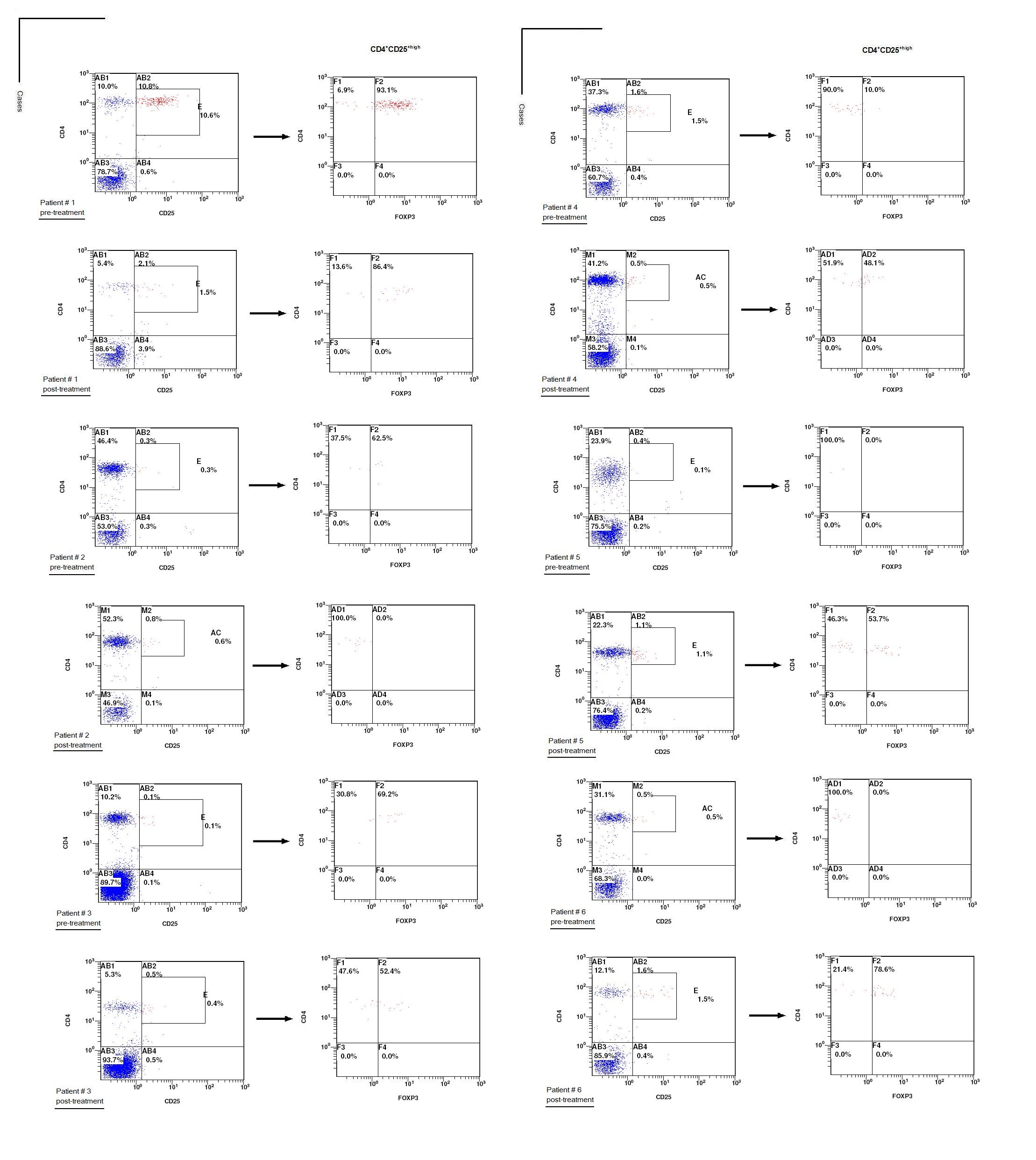
**

**Supplementary Figure 6** Histogram data of 6 patients for comparison of levels of CD4^+^CD25^+high^ and CD4^+^CD25^+high^Foxp3^+^ T cells before and after immunomodulatary treatment. Patient # 1 had overinduced CD4^+^CD25^+high^ and CD4^+^CD25^+high^Foxp3^+^ T cells before treatment. After treatment, both of them were effectively downmodulated to low levels. The other 5 cases had low levels of CD4^+^CD25^+high^ and CD4^+^ CD25^+high^Foxp3^+^ T cells initially. There was no significant influence of the treatment to the levels of the two T cell subpopulations.
